# ARFGAP1 Is Dynamically Associated with Lipid Droplets in Hepatocytes

**DOI:** 10.1371/journal.pone.0111309

**Published:** 2014-11-14

**Authors:** Joan Gannon, Julia Fernandez-Rodriguez, Hussam Alamri, Shi Bo Feng, Fariba Kalantari, Sarita Negi, Amy H. Y. Wong, Alexander Mazur, Lennart Asp, Ali Fazel, Ayat Salman, Anthoula Lazaris, Peter Metrakos, John J. M. Bergeron, Tommy Nilsson

**Affiliations:** 1 The Research Institute of the McGill University Health Centre, McGill, Royal Victoria Hospital, 687 Pine Avenue West, Montreal, Quebec, Canada; 2 The Centre for Cellular Imaging at the University of Gothenburg, Gothenburg, Sweden; 3 The Department of Medical and Clinical Genetics, Institute of Biomedicine, University of Gothenburg, Gothenburg, Sweden; National Cancer Institute, United States of America

## Abstract

The ARF GTPase Activating Protein 1 (ARFGAP1) associates mainly with the cytosolic side of Golgi cisternal membranes where it participates in the formation of both COPI and clathrin-coated vesicles. In this study, we show that ARFGAP1 associates transiently with lipid droplets upon addition of oleate in cultured cells. Also, that addition of cyclic AMP shifts ARFGAP1 from lipid droplets to the Golgi apparatus and that overexpression and knockdown of ARFGAP1 affect lipid droplet formation. Examination of human liver tissue reveals that ARFGAP1 is found associated with lipid droplets at steady state in some but not all hepatocytes.

## Introduction

Both genetics- and cell biology-based experiments link COPI coat components including the small GTPase, ARF1, coatomer and the exchange factor, GBF1 to lipid droplet biogenesis [1]–[5]. ARF1 is one of 5 ARFs associated with the Golgi apparatus (for review, see [6]) and through GTP hydrolysis catalyzed by an ARF GTP Activating Protein (ARFGAP), regulates COPI coat formation [7]–[9] and sorting of proteins into COPI vesicles [10]–[12]. ARF1 is also linked to recruitment of lipid modifying enzymes such as phospholipase D (PLD1) [13], [14] and phosphoinositol kinases [15] to Golgi membranes as well as GGA proteins involved in clathrin- coated vesicle formation from Golgi membranes [16], [17]. In addition, ARF1 regulates association of adipocyte differentiation-related protein (ADRP, PLIN2) and PLD1 on the cytosolic leaflet of lipid droplets [18], [19]. ARF1 function is regulated by the exchange of bound GDP for GTP via an exchange factor (e.g. GBF1) transitioning ARF1 from an inactive to an active conformation. This is then followed by hydrolysis of bound GTP to GDP transitioning ARF1 back to an inactive conformation. Due to its intrinsically low GTP hydrolysis rate, ARF1 requires an activating protein for GTP hydrolysis. In mammalian cells, three related activating proteins termed ARFGAP1-3 are linked to COPI vesicle formation through ARF of which ARFGAP1 is the prototypical protein. Of the three, ARFGAP1 appears sensitive to both membrane lipid composition [20], [21] and membrane curvature [22], [23] and has also been proposed to act as a structural component alongside coatomer to form the COPI coat [24], [25]. In addition, ARFGAP1 has been shown to regulate AP-2-dependent endocytosis through the binding of adaptor proteins [26] as well as LRRK2 linked to sporadic and autosomal dominant forms of Parkinson's disease [27], [28]. In this study, we show that ARFGAP1 is present on lipid droplets and that it appears important for lipid droplet formation. Lipid droplet association of ARFGAP1 is observed transiently in cultured cells and at steady state in some but not all hepatocytes of human liver tissue.

## Results

### Overexpression or knockdown of ARFGAP1 affect lipid droplet size and numbers

**Figure 1:**
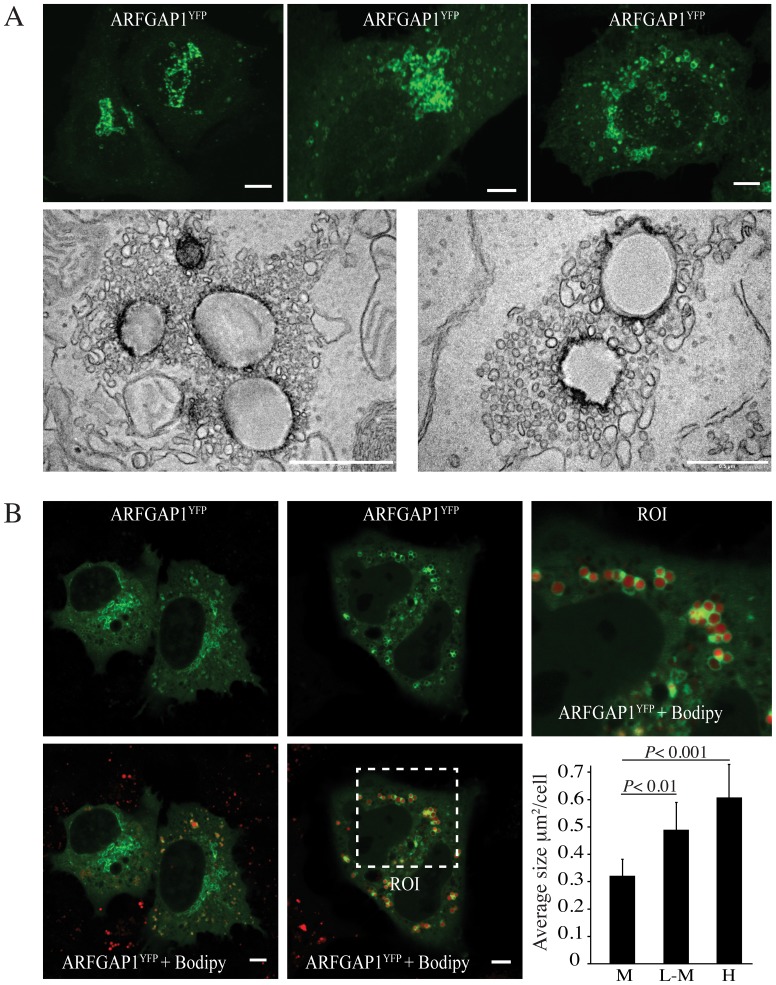
Overexpression of ARFGAP1 promotes lipid droplet formation. In A, HeLa cells were transfected with plasmid DNA encoding ARFGAP1 fused to EYFP (ARFGAP1^YFP^) [47], fixed and imaged at 24 hours post transfection. Representative fields were captured with cells expressing relatively low (upper left panel) or high (upper middle and right panels) levels of the fusion protein. Bars in upper panels  = 5µm. Epon-embedded transfected cells were also examined at the ultrastructural level through transmission electron microscopy (see Materials and Methods). Bars in lower left and right panels from electron micrographs correspond to 1µm and 0.5µm, respectively. In B, HepG2 cells were transfected with plasmid DNA encoding ARFGAP1^YFP^ as above and counterstained with Bodipy 558/568C_12_ to reveal lipid droplets. Representative images are shown of cells with low (left two panels) and high expression levels (middle two panels). The upper right panel shows an enlarged region of interest (ROI). Bars  = 5µm. The average size of Bodipy-stained structures was quantified in cells with low to moderate (L–M) and high expression levels of ARFGAP1^YFP^ as well as in Mock (M) transfected cells. *P*-values were obtained using the Mann–Whitney rank sum test comparing the average size of Bodipy-stained lipid droplets in Mock transfected cells with those in cells of low to moderate (*P*<0.01) or high expression (*P*<0.001) of ARFGAP1^YFP^.

In previous work examining the role of ARFGAP in vesicle budding and scission in the Golgi apparatus of HeLa cells [20], we noted that upon high over-expression, ARFGAP1 fused to enhanced yellow fluorescence protein (ARFGAP1^YFP^) localized to ring-like structures suggestive of lipid droplets (Fig. 1A, upper right panel) rather than the Golgi apparatus (Fig. 1A, upper left panel). Some over-expressing cells also revealed an intermediate pattern with some ring-like structures positive for ARFGAP1^YFP^ in addition to the juxta-nuclear Golgi apparatus (Fig. 1A, upper middle panel). When examined at the ultra structural level, lipid droplet-like structures were observed surrounded by extensive vesicular/tubular profiles (Fig. 1A, lower panels). Overexpression of ARFGAP1 is known to affect the integrity of the Golgi apparatus resulting in a brefeldin A-like phenotype [29] and it is possible that observed vesicular/tubular profiles correspond to Golgi remnants. Another possibility is that such vesicular/tubular profiles represent smaller lipid droplets and that ARFGAP1 is engaged in trafficking between such smaller lipid droplets and larger ones. ARFGAP1^YFP^ fluorescence was observed both adjacent to and around lipid droplet-like structures (see also Figure S1 detailing the movement of ARFGAP1^YFP^ fluorescent structures over the course of 40 minutes). To confirm that observed structures were lipid-based, we transfected plasmid DNA encoding ARFGAP1^YFP^ into cultured HepG2 human hepatoma cells and counter-stained with Bodipy 558/568 C_12_ to stain neutral lipids. As can be seen in the upper and lower middle panels of Figure 1B and in the enlarged region of interest (ROI), ARFGAP1^YFP^ localized to and near ring-like structures surrounding neutral lipid material when expressed at high levels as compared to cells expressing low levels of ARFGAP1^YFP^ (Fig. 1B upper and lower left panels). The average size of Bodipy-stained lipid droplets was measured in 250 cells/experiment in 3 independent experiments (see Materials and Methods). Quantification (Fig. 1B-lower right) revealed a two-fold increase in total lipid droplet area in cells with high (H) expression compared to mock (M) transfected cells. Even cells with low to moderate (L–M) expression revealed an increase in the area of lipid droplet-like structures staining positive for Bodipy 558/568 C_12_. This suggests that over-expression of ARFGAP1^YFP^ promotes lipid droplet formation in both HeLa and HepG2 cells. Note that in contrast to HeLa cells, HepG2 cells have a basal level of lipid droplets.

**Figure 2:**
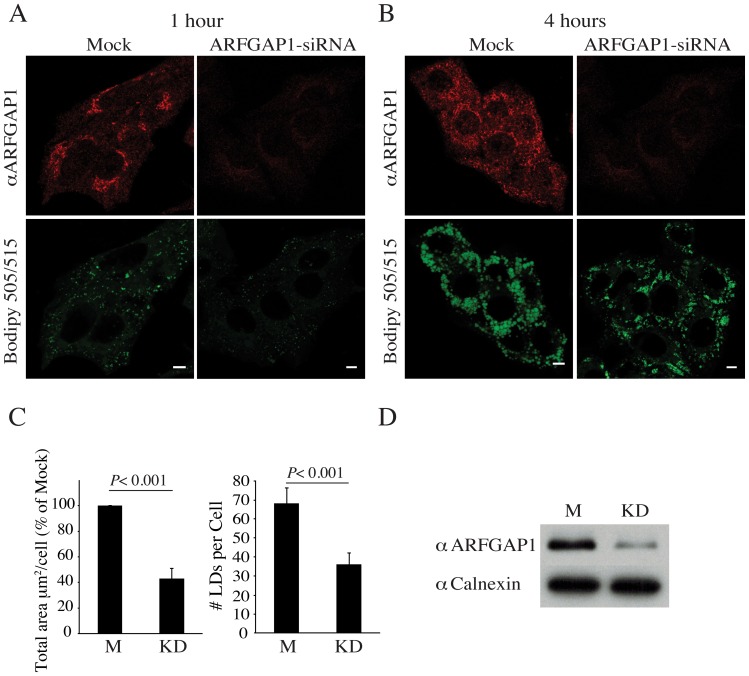
Knockdown of ARFGAP1 affects lipid droplet formation. Shown in A and B are HepG2 cells transfected with siRNA specific for ARFGAP1 or with a scrambled sequence (Mock) and 24 hours post transfection, incubated with 0.5 mM oleate to promote lipid droplet formation. Cells were then fixed 1 hour or 4 hours after addition of oleate and stained to reveal endogenous ARFGAP1 (red) and lipid droplets (green) using a rabbit polyclonal to ARFGAP1 and Bodipy 493/503, respectively. Bars  = 5µm. In C, left, lipid droplets per cell were quantified as total area of Bodipy493/503 stained droplets per cell expressed as a percentage of the total area of Bodipy493/503 stained droplets in cells transfected with siRNA specific to ARFGAP1 compared to Mock transfected cells. In C, right, the number of Bodipy493/503 stained lipid droplets per cell was compared between cells transfected with siRNA specific to ARFGAP1 and Mock-transfected cells. P-values were obtained using the Mann–Whitney rank sum test comparing the number (*P*<0.001) or average size (*P*<0.001) of Bodipy-stained lipid droplets in Mock transfected cells with those of transfected cells. In D, western blots of ARFGAP1 and calnexin. Extracts from cells transfected either with scrambled siRNA (Mock, M) or siRNA specific to ARFGAP1 for knockdown (KD) were subjected to SDS-PAGE, transfer to PVDF membranes and blotted against ARFGAP1 and Calnexin using specific antibodies.

We next examined whether knockdown of endogenous ARFGAP1 affects lipid droplet formation in HepG2 cells. siRNA specific for endogenous ARFGAP1 causing knockdown (KD) or siRNA composed of a scrambled sequence serving as a mock (M) control were transfected into HepG2 cells. At 24 hours post transfection, cells were incubated with oleate (final concentration at 0.5 mM) for either 1 or 4 hours. After exposure to oleate, cells were fixed and processed for indirect immunofluorescence using an antibody specific to ARFGAP1 [30]. In mock transfected cells incubated with oleate for 1 hour, this antibody gave rise to a juxta nuclear ribbon-like structure consistent with the Golgi apparatus (Fig. 2A, upper left panel) whilst in cells transfected with siRNA specific for ARFGAP1, only weak staining was observed consistent with knockdown of ARFGAP1. Under these conditions, lipid structures staining positive for Bodipy 493/503 decreased both in area per cell and number per cell (Fig. 2 C). Total area of Bodipy-staining of lipid droplets in each cell as well as the number of stained lipid droplets per cell was quantified in 150 cells per experiment in five independent experiments using ImageJ 1.45 s (see Image Analysis in Materials and Methods). About a 60% and 45% reduction in the area and number of Bodipy positive structures were observed upon knockdown of ARFGAP1, respectively (Fig. 2C). The difference between mock transfected cells and cells transfected with siRNA specific for ARFGAP1 was less pronounced after 4 hours of incubation with oleate with respect to number of observed droplet-like structures staining positive for Bodipy (Figure 2B). Instead, we observed a significant decrease in size of lipid droplets produced in cells where ARFGAP1 expression had been diminished (compare the two lower panels of Fig. 2B). In addition, we noted in mock transfected cells after 4 hours of incubation of oleate, that there was an apparent increase in cytoplasmic staining of endogenous ARFGAP1 (Fig. 2B upper left panel) in close proximity to structures staining positive for Bodipy. That siRNA specific for ARFGAP1 caused knockdown of endogenous ARFGAP1 was further examined by western blotting. Figure 2D shows that cells transfected with siRNA specific for ARFGAP1 to cause knockdown (KD) have a marked decrease in the antibody-generated reaction product specific for ARFGAP1 compared to that of mock (M) transfected cells. The ER-resident chaperone calnexin was monitored as a control using an antibody specific for its cytoplasmic domain [31]. Thus, decreased staining of ARFGAP1 as deduced by indirect immunofluorescence and western blotting suggest that the majority of ARFGAP1 had been knocked down. We conclude from this that lowering expression of endogenous ARFGAP1 affects lipid droplet formation. Also, that exposure to oleate for 4 hours affects localization of ARFGAP1.

### ARFGAP1 is transiently recruited to lipid droplets in HepG2 cells upon addition of oleate

**Figure 3:**
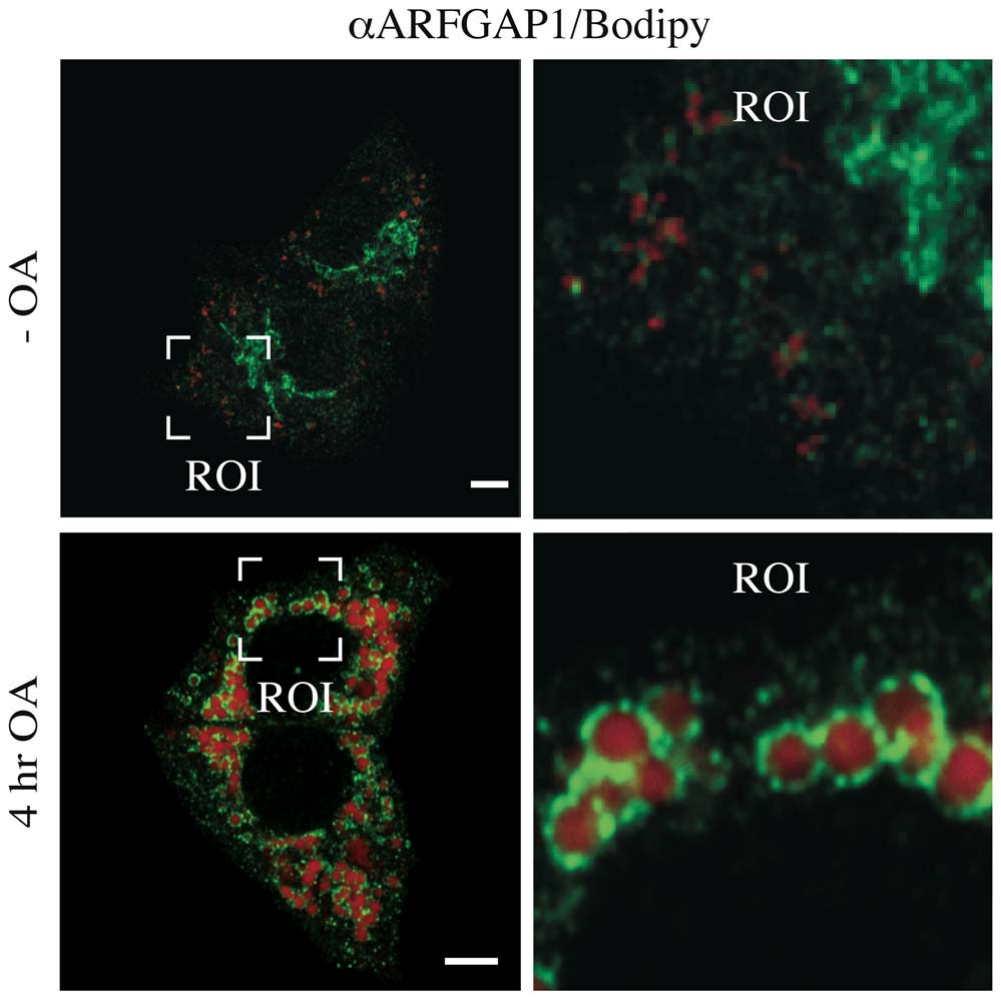
ARFGAP1 relocates from the Golgi apparatus to lipid droplets upon addition of oleate. HepG2 cells were fixed and processed for indirect immunofluorescence before (-OA) and 4 hours after addition of 0.5 mM oleate and stained to reveal endogenous ARFGAP1 (green) and lipid droplets (red) using a rabbit polyclonal to ARFGAP1 and Bodipy 558/568 C_12_, respectively. Bars  = 5µm.

**Figure 4:**
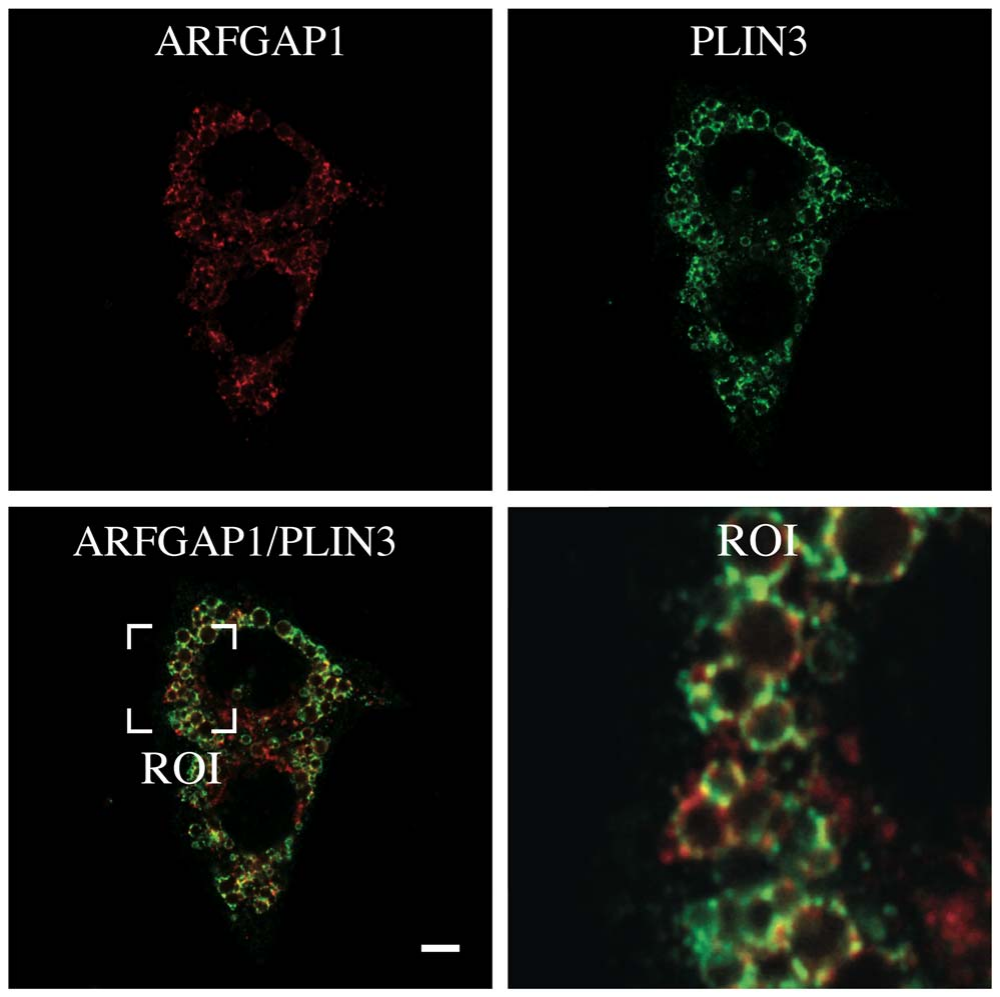
ARFGAP1 co-localizes with PLIN3 on lipid droplets. Endogenous ARFGAP1 staining (red) was compared with antibody staining of endogenous PLIN3 (green). An enlarged region of interest (ROI) is shown in the bottom right panel of the merged image (ARFGAP1/PLIN3). Bar  = 5µm.

**Figure 5:**
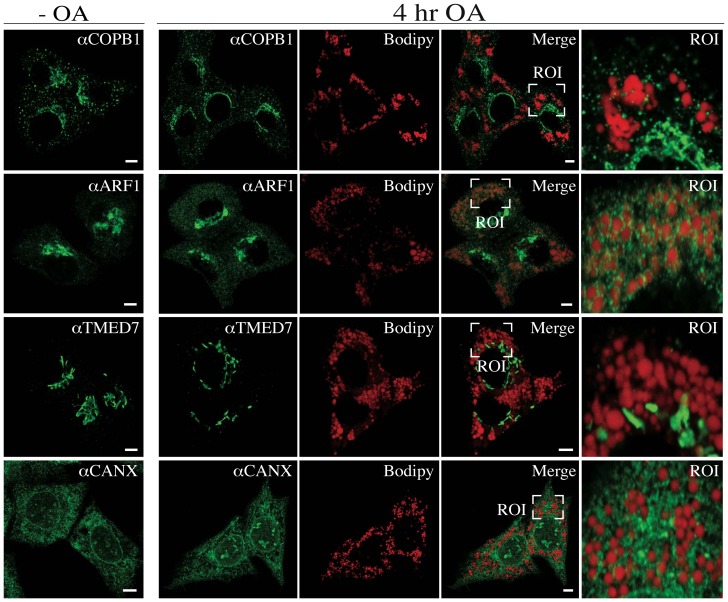
Lipid droplet localization of ARFGAP1 is distinct from other endogenous Golgi and ER markers. Indirect immunofluorescence of COPB1 in HepG2 cells, ARF1 in McA-RH7777 cells, TMED7 and CANX in HepG2 cells before (4 right panels) and 4 hours after addition of 0.5 mM oleate. Enlarged regions of interest are shown in the 4 left panels. Bars  = 7µm.

To investigate the localization of ARFGAP1 upon oleate addition more precisely; we fixed and processed HepG2 cells for indirect immunofluorescence both before and 4 hours after addition of 0.5 mM oleate (final concentration). As shown in Figure 3 (upper panels), there was no apparent co-localization between endogenous ARFGAP1 and structures staining positive for Bodipy before addition of oleate. In contrast, after 4 hours of incubation with oleate, most of the ARFGAP1 staining was seen surrounding Bodipy-positive lipid droplets (Fig. 3 lower panels). The staining for ARFGAP1 was compared with a known lipid droplet marker, PLIN3 (Perilipin 3/TIP47) which has also been implicated in Golgi/endosomal trafficking [32]. As shown in Figure 4 and in the enlarged region of interest (ROI), there was considerable co-localization (yellow) between ARFGAP1 (red) and PLIN3 (green). This shows that ARFGAP1 localizes to lipid droplet structures 4 hours after oleate addition partly overlapping with PLIN3. As ARFGAP1 was first identified as an activator protein for the small GTPase ARF1 in the context of coat regulation of COPI coatomer responsible for the generation of COPI transport vesicles and retrograde transport in the Golgi apparatus (for review, see [32]), it was of further interest to test whether these and other Golgi markers also localized to lipid droplet structures upon addition of oleate. Figure 5 row of upper panels show the distribution of COPB1 (βCOP), a component of the 7 subunit coatomer complex as revealed by the mouse monoclonal antibody CM1A10 specific for one of its components [33] before (-OA) and 4 hours after addition of oleate (4 hr OA). Most staining forCOPB1 appeared as juxta nuclear Golgi staining with additional punctate staining throughout the cytoplasm, a staining pattern typical of coatomer. No apparent co-localization with lipid droplets was observed under these conditions. ARF1 was probed for in the hepatoma cell line, McA-RH7777 using a rabbit polyclonal (Fig. 5, αARF1) [30] resulting in a predominant juxta nuclear Golgi staining with occasional small punctate structures throughout the cytoplasm of cells before addition of oleate. At 4 hours after addition of oleate, staining remained predominantly juxta nuclear with an increase in staining of cytoplasmic structures. A minor portion of such cytoplasmic structures appeared close to lipid droplets as revealed by Bodipy stain. It is likely that the antibody used here recognizes additional ARFs due to their close homology. From above experiments, it appears that ARFGAP1 distributes to lipid droplets upon addition of oleate whereas coatomer and most of ARF1 do not. At 4 hours after addition of oleate, the Golgi apparatus appears intact. This was confirmed in HepG2 cells using an antibody to TMED7 (gp27) (Fig. 5, αTMED7), a member of the gp25L/emp24 family of small transmembrane proteins of the early secretory pathway [34]. Exclusive juxta nuclear Golgi staining was observed before and 4 hours after addition of oleate. An antibody to the cytoplasmic domain of CANX (calnexin) was used to test for changes of the ER in HepG2 cells 4 hours after addition of oleate. No discernable difference was observed (Fig 5, αCANX). This shows that the gross architecture of the Golgi apparatus or the ER is not affected at 4 hours after addition of oleate despite loss of ARFGAP1 to lipid droplets.

**Figure 6:**
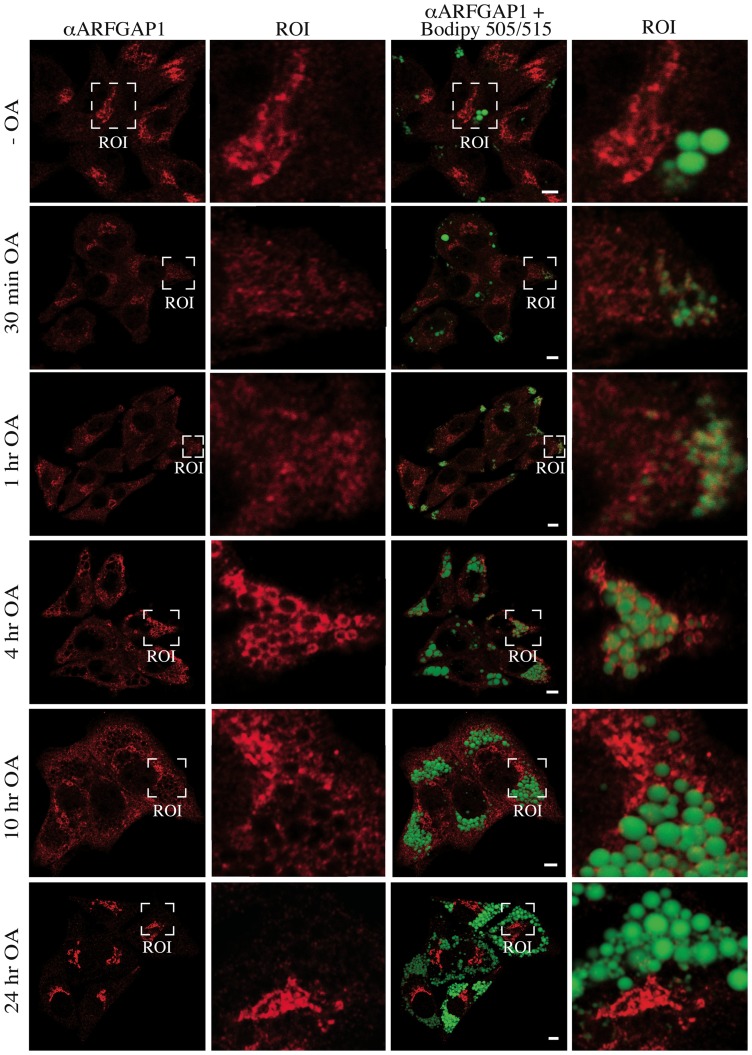
ARFGAP1 association with lipid droplets upon oleate addition is transient. Indirect immunofluorescence of endogenous ARFGAP1 (red) in McA-RH7777 cells before and after incubation with oleate for various lengths of time. Lipid droplets were stained using Bodipy 493/503 (green). Enlarged regions of interest (ROI) are displayed on the right of each image. Bars  = 5µm.

**Figure 7:**
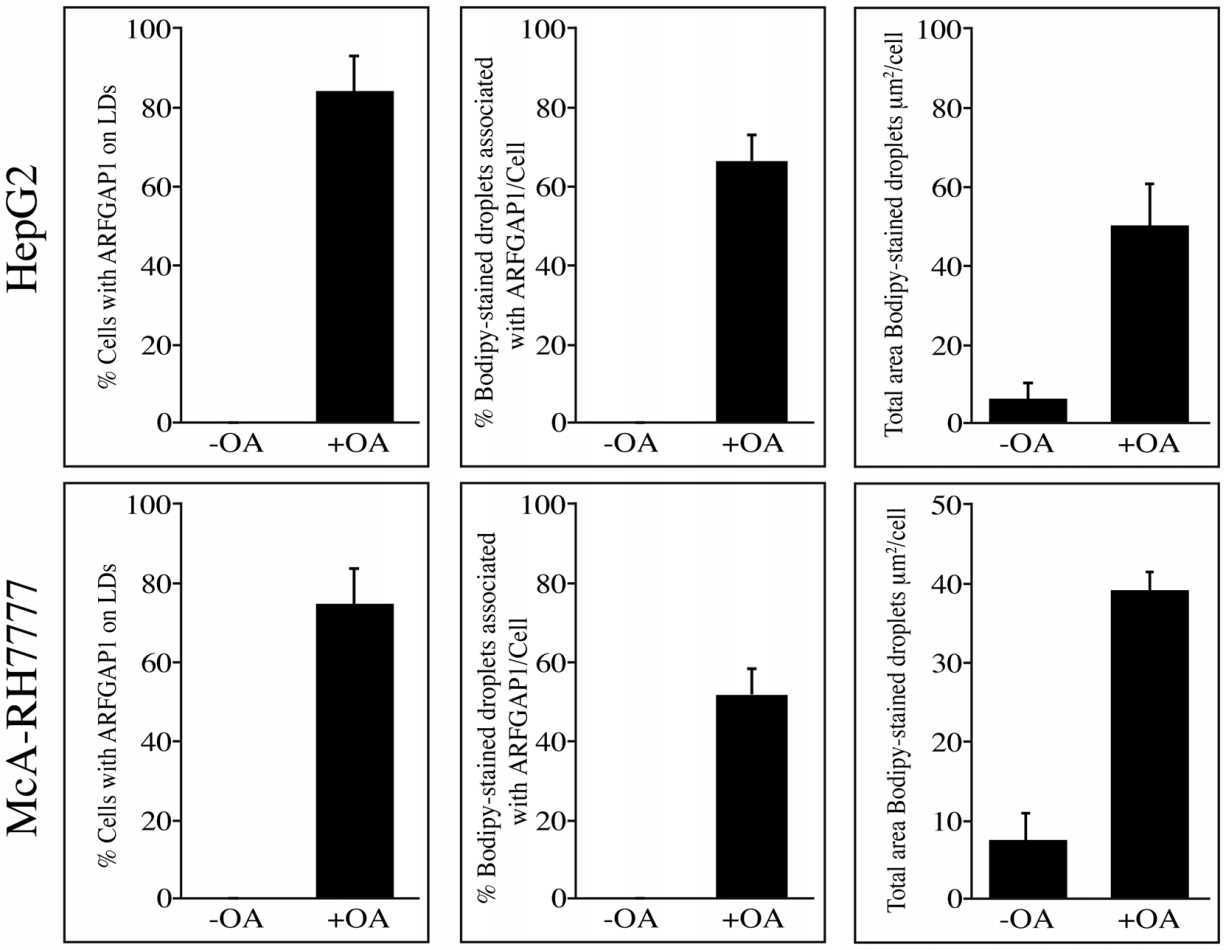
Quantification of ARFGAP1 associated with lipid droplets . Endogenous ARFGAP1 as revealed by indirect immunofluorescence was quantified before (-OA) and 4 hours after addition of oleate (+OA) in HepG2 and McA-RH7777 cells expressed as % cells with ARFGAP1 staining (as revealed through indirect immunofluorescence) or % Bodipy-stained droplets associated with ARFGAP1 per cell. On the right, total area of Bodipy-stained lipid droplets per µm^2^ per cell.

**Figure 8:**
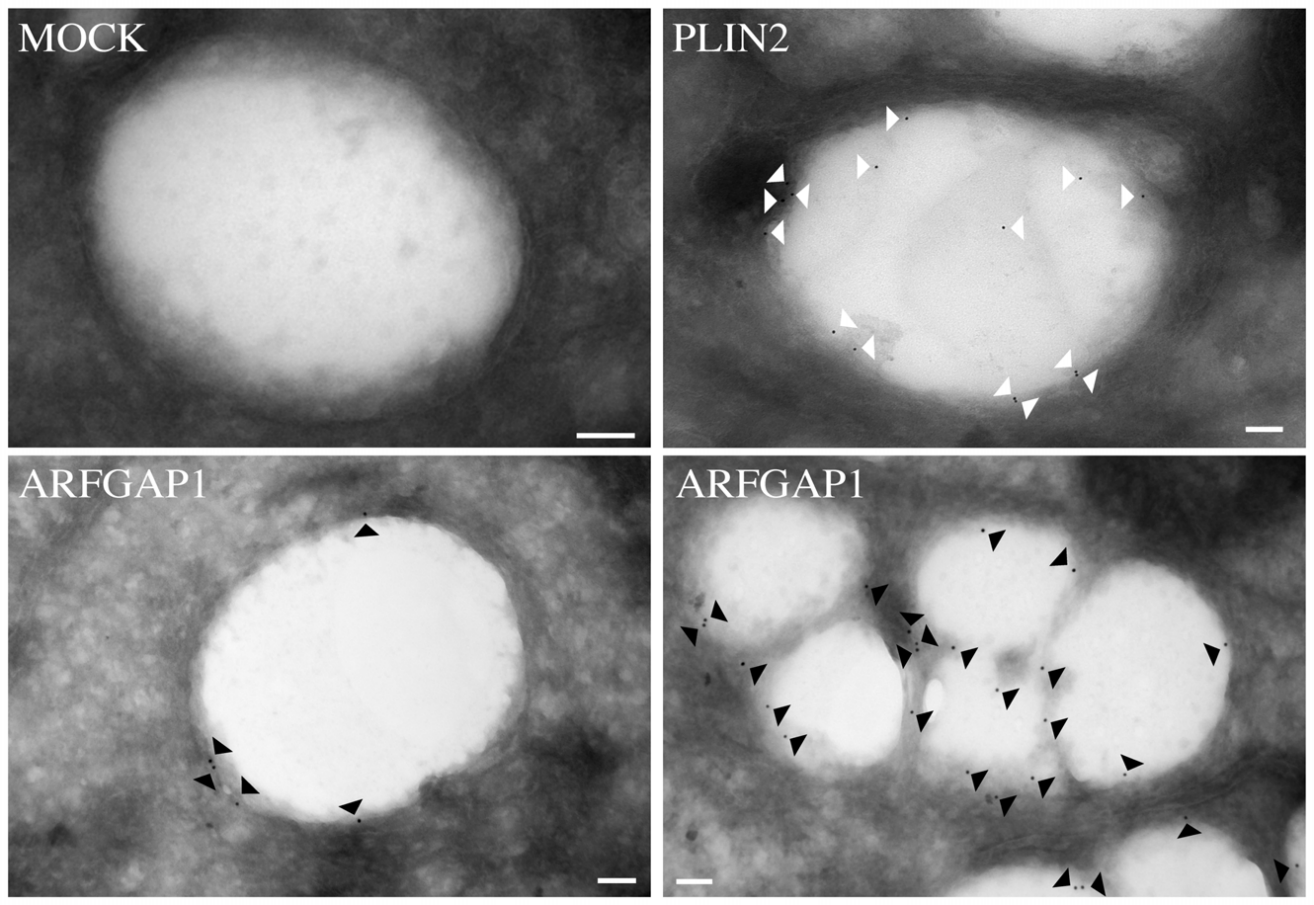
ARFGAP1 localizes to lipid droplets as revealed by immuno-gold transmission electron microscopy. Thin frozen sections of HepG2 cells fixed 4 hours after addition of 0.5 mM oleate were incubated individually with antibodies to either PLIN2 (white arrowheads) or ARFGAP1 (black arrowheads) followed by gold-conjugated secondary antibodies to reveal endogenous proteins at the ultra structural level. Bars  = 100 nm.

To investigate when ARFGAP1 redistributes to lipid droplet structures, McA-RH7777 cells were incubated with oleate for various lengths of time (Fig. 6). This revealed that though most of ARFGAP1 localized to lipid droplets 4 hours after addition of oleate, much less was observed at later time points suggesting a transient association. At 10 hours after oleate addition, over half of ARFGAP1 still remained associated with lipid droplet structures whereas after 24 hours, ARFGAP1 was exclusively located at the juxta nuclear Golgi area. The shift in distribution of endogenous ARFGAP1 from the Golgi apparatus to lipid droplets was quantified 4 hours after addition of oleate in 350 cells per experiment in 5 independent experiments using ImageJ 1.45 s software (see Materials and Methods). In HepG2 and McA-RH7777 cells, the percentage of cells that had ARFGAP1 associated with lipid droplets increased from 0% to 80% and 0% to 75% at 4 hours after addition of oleate, respectively (Fig. 7-left). About 65% and 50% of Bodipy-stained structures were associated with ARFGAP1 per cell at 4 hours after oleate addition in HepG2 and McA-RH7777 cells, respectively (Fig. 7, middle). Addition of oleate caused a marked increase in the area of Bodipy-positive lipid droplets corresponding to a 10 and 5 fold increase in HepG2 and McA-RH7777 cells, respectively (Fig. 7, right). To confirm that ARFGAP1 indeed localizes to lipid droplets upon addition of oleate, HepG2 cells were fixed and processed for immuno-gold transmission electron microscopy (see Materials and Methods). Figure 8 shows single antigen gold-labeled thin frozen sections after 4 hours incubation with oleate incubated with a control antibody (MOCK), a goat polyclonal antibody to ADRP (PLIN2), a lipid droplet marker, or a rabbit polyclonal antibody to ARFGAP1 [30]. Arrowheads in black and white point to gold particles detected revealing staining at the periphery of lipid droplets of both ADRP and ARFGAP1. On occasion, gold particles for ADRP were detected also within the lipid droplet structure.

### Lipid droplet association of ARFGAP1 is reversed upon addition of cAMP

**Figure 9:**
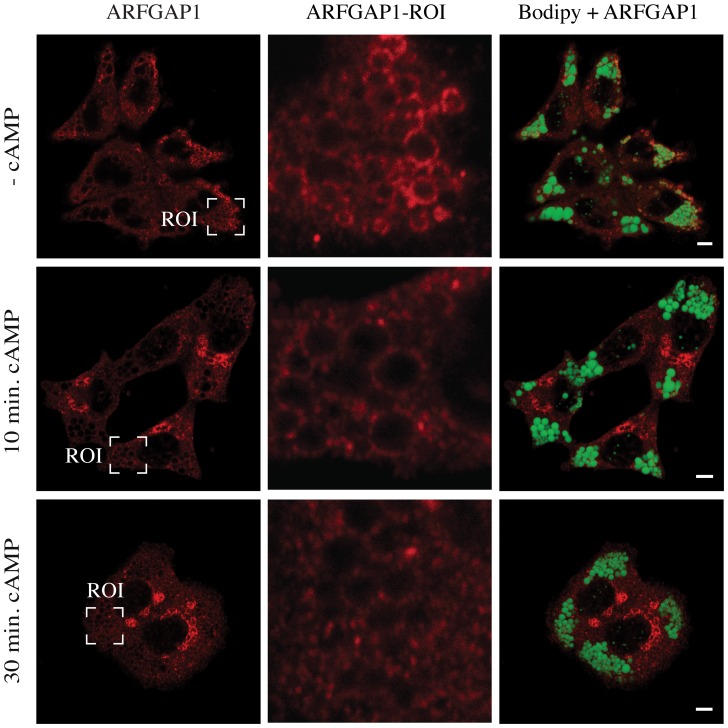
Cyclic AMP reverses lipid droplet association of ARFGAP1. McA-RH7777 cells were incubated with 0.5 mM oleate for 4 hours after which cyclic AMP (cAMP) was added to a final concentration of 37.5µg/ml. The cells were simultaneously stained for endogenous ARFGAP1 (red) and neutral lipid with Bodipy 493/503 (green). Bars  = 5µm.

Based on above data, it appears that most if not all ARFGAP1 associates transiently with lipid droplets peaking at 4 hours after addition of oleate. Such transient association suggests a dynamic recruitment of ARFGAP1 to lipid droplets upon oleate uptake, possibly regulated by cAMP effector proteins such as cAMP-dependent protein kinase (PKA) shown to affect lipid homeostasis [35]. To test this, McA-RH7777 cells were incubated in the presence of oleate for 4 hours (Fig. 9) followed by addition of cAMP. Before addition of cAMP, most if not all of endogenous ARFGAP1 localized to peri-lipid droplet structures positive for Bodipy (Fig. 9 upper panels). As above, no discernable juxta nuclear Golgi staining was observed. At 10 minutes after cAMP addition, staining for endogenous ARFGAP1 was detected in the juxta nuclear Golgi region as well as around lipid droplet structures (Fig. 9-middle panels). At 30 minutes after cAMP addition, staining for endogenous ARFGAP1 was mainly detected in the juxta nuclear Golgi apparatus with some staining remaining around lipid droplet structures. At these time points (10 and 30 minutes), no discernable decrease in the overall amount of lipid droplets was observed (Fig. 9).

### ARFGAP1 associates with lipid droplets in human liver

**Figure 10:**
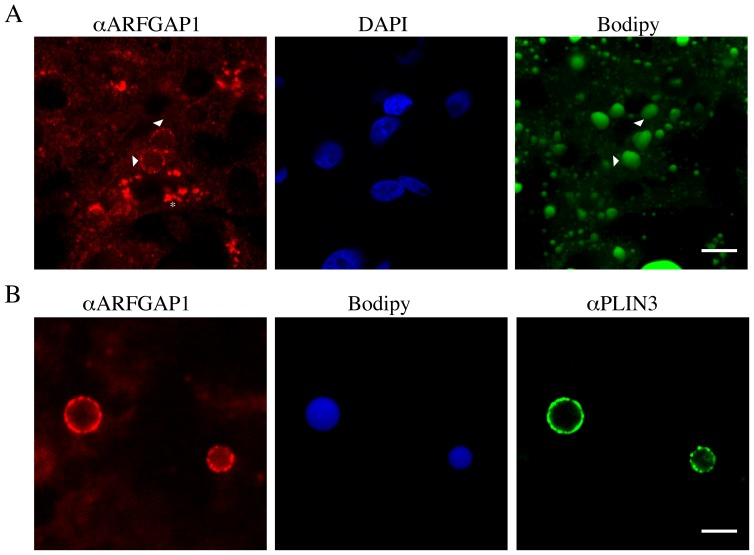
ARFGAP1 associates with lipid droplets at steady state in human livers. In A and B, thin frozen sections of two human livers were fixed and processed for indirect immunofluorescence as described in Materials and Methods. Endogenous ARFGAP1 was revealed as in Figure 4. Nuclear DNA was in A stained with DAPI and lipid droplets in A and B with Bodipy 493/503. Bar  = 10µm. In B, endogenous ARFGAP1 and PLIN3 were revealed as in Figure 4. Pseudo colors were assigned to each panel. Bar  = 15µm.

Though it appears that ARFGAP1 localizes transiently to lipid droplets structures at 4 hours after addition of oleate in both McA-RH7777 and HepG2, it was important to rule out that such a shift in steady state distribution reflects an unphysiological condition induced by a sudden influx of oleate. We therefore examined ARFGAP1 in human hepatic tissue. Thin frozen sections were generated from liver tissue resected from patients undergoing surgery for removal of metastases of the liver as well as from donor livers obtained through Transplant Quebec (http://transplantquebec.ca/en) (see Materials and Methods) and subjected to indirect immunofluorescence. Figure 10A shows a frozen section stained for endogenous ARFGAP1 (αARFGAP1, left panel), DAPI to reveal nuclear chromatin (middle panel) and Bodipy 493/503 to stain for neutral lipids (right panel). Some of the staining for ARFGAP1 decorates what appear to be large lipid droplet structures often comparable or larger in size than the nucleus. In this section, ARFGAP1 is seen both as a compact Golgi-like staining as well as decorating lipid droplet-like structures (arrowheads). The staining of endogenous ARFGAP1 was also compared with the lipid droplet marker protein PLIN3 and Bodipy 493/503 to stain for neutral lipids (Fig. 10B-right panel). Note that staining of endogenous ARFGAP1 (Fig. 10B-left panel) mostly but not fully overlap with that of PLIN3 (Fig. 10B-right panel). In liver sections examined, overlapping staining between ARFGAP1 and PLIN3 approached 60% based on 138 lipid droplets. However, the appearance of ARFGAP1 on lipid droplet structures in a given liver section is not uniform nor is the overall frequency of ARFGAP1 positive lipid droplet structures when compared between livers of different patients/donors. Nevertheless, the appearance of ARFGAP1 on lipid droplet structures suggests a physiological relevance prompting further studies.

## Discussion

Previous proteomics data has included ARFGAP1 as a potential lipid droplet–associated protein under conditions where GTP hydrolysis was inhibited through addition of the non-hydrolyzable GTP analogue, GTPγS [36]. More recent data based on interference RNA-based experiments suggests that ARFGAPs 1-3 promote lipid droplet formation in HeLa cells [37]. In that study, similar effects were also observed upon knockdown of COPB1, GBF1 and class II ARFs (ARF4 and ARF5). The authors further concluded, based on indirect immunofluorescence data, that COPB1, GBF1, ARF4 and ARFGAP1 do not associate with lipid droplets, at steady state [37]. This is in contrast to recent studies suggesting a role for COPI components in establishing/maintaining interconnections between lipid droplets and the ER as well as in the formation of vesicles budding of the surface of the lipid droplet [4], [5]. Our study shows that ARFGAP1 associates transiently with lipid droplets in cultured hepatoma cells (HepG2 and McA-RH7777) upon addition of oleate and at steady state on lipid droplets of some but not all hepatocytes of human liver. Indirect immunofluorescence staining of endogenous ARFGAP1 or fluorescence from expressed ARFGAP1^YFP^ is clearly visible as peri-droplet staining but also on adjacent juxta-posed structures. Though immuno-gold labeling of ARFGAP1 show gold particles decorating the perimeter of the lipid droplet, we cannot formally rule out that such gold particles label ARFGAP1 on structures that reside very close to the surface of the lipid droplet (the combined size of primary and secondary antibodies gives rise to an uncertainty in distance of up to 30nm between the antigen and the gold particle).

Based on the evidence at hand, we suggest that ARFGAP1 association with lipid droplets of hepatocytes of human liver indicates a physiological and functional context between ARFGAP1 and lipid droplets. Indeed, over expression of ARFGAP1 promotes lipid droplet formation in both HeLa and HepG2 cells whereas knockdown experiments of ARFAGP1 impair lipid droplet formation in both HepG2 and McA-RH7777 cells. It is possible that over-expression of ARFGAP1 causes cellular stress and that stress in turn promotes lipid droplet formation [38] independent from ARFGAP1 function. Such stress could either be through its catalytic activity or lipid-binding properties. As such, we interpret our over-expression results with caution. It is further possible that knockdown experiments cause cellular stress. However, the phenotype is here the opposite in that knockdown of ARFGAP1 impairs lipid droplet formation. As components of the COPI machinery have been linked to lipid droplet biogenesis, the phenotype observed may be explained through an impaired Golgi-derived delivery of components needed for lipid droplet formation [1]–[3], maintenance of LD-ER connections and/or formation of lipid-droplet derived vesicles [4], [5]. It is further possible that addition of oleate results in cellular stress and that this prompts a transient relocation of ARFGAP1 to lipid droplets. However, even though relocation is transient, addition of cyclic AMP reverses ARFGAP1 back to the Golgi apparatus indicating and underlying physiological process. This is supported by the observation that ARFGAP1 localizes to lipid droplets at steady state in some but not all hepatocytes of human livers. As these livers all have varying degrees of steatosis, it will be of interest in the future to determine whether ARFGAP1 localization to lipid droplets correlates with certain pathophysiological states. In conclusion, we have demonstrated that ARFGAP1 associates transiently or at steady state with lipid droplets and to structures in close juxtaposition to lipid droplets in cell culture and human liver tissue, respectively, and that ARFGAP1 affects lipid droplet formation. The physiological context as to why ARFGAP1 is present on lipid droplets remains to be determined. As ARFGAP1 is historically linked to COPI vesicle function by serving as an activating protein for ARF1 in the recruitment of coatomer as well as in the scission of vesicles, an involvement in COPI function at the level of lipid droplet biogenesis and homeostasis is possible [4], [5]. Other putative functions include binding to SNARE proteins present on lipid droplets involved in membrane fusion [39] or in the regulation of motility of lipid droplets through ARF1 [40]. It is also conceivable that ARFGAP1 acts on ARF1 in the recruitment of phospholipid-modifying enzymes [13]–[15] or PLIN2 [18], [19] to the surface of lipid droplets or that its presence on lipid droplets signifies a function yet to be determined.

## Materials and Methods

### Chemicals and Reagents

Phosphate buffered saline (PBS), Tween (0.05%), oleate, 8-Bromoadenosine-3′, 5′-cyclic monophosphate (c-AMP) sodium salt, Nile red stain, Coomassie Brilliant Blue G, sodium fluoride, EDTA, Tris-HCl, sucrose, tricine, NaCl, Na_2_HPO_4_, KH_2_PO_4_, CaCl_2_, MgCL_2_, Tween 20, Fish skin gelatin, Saponin, PFA, Mowiol, ammonium choride, lead citrate, bromophenol blue, Biomax X-Omat XAR or MR films from Eastman Kodak, 10 nm colloidal gold-affinity purified rabbit anti-Goat, ACN, gelatin, formic acid, and sodium citrate were all from Sigma-Aldrich (Oakville, Ontario, Canada). Acetone, PVDF, KCl, water (LCMS grade), DMSO and trypsin were from Fisher Scientific (Quebec, Canada). FuGENE HD Transfection Reagent, SDS and Tris base were from Roche Ltd (Laval, Qc, Canada). Sodium cacodylate and Epon 812 were from Mecalab Ltd (Quebec, Canada). DTT was from Biomol (Hamburg, Germany). ECL detection kit was from GE Healthcare (Baie d'Urfe, Quebec, Canada). Thirty percent (wt/vol) acrylamide/0.8% (wt/vol) bis-acrylamide solution was from Bio-Rad (Mississauga, On. Canada). Uranyl acetate, glutaraldehyde, and glycerol were from Merck (Kirkland, Qc, Canada). Osmium tetroxide was from Agar Scientific (Essex, United Kingdom). Laemmli 2 X from Bio-Rad (Ontario, Canada). ProLong Gold Antifade, MEM, GIBCO DMEM, FBS, penicillin streptomyocin, Bodipy 493/503, Bodipy 558/568 C12, glutamine, Lipofectamine siRNA was purchased from Invitrogen (Carlsbad, CA). 12 nm colloidal gold-affiniPure Goat anti-Rabbit IgG (H+L) was purchased from Cedar Lane laboratories (Ontario, Canada). ARFGAP1-EYFP was kindly supplied by Dr. J. Lippincott-Schwartz (National Institutes of Health, Bethesda, MD). The mouse monoclonal antibody CMIA10 against COPB1 and rabbit polyclonal antibodies to ARFGAP1 ARF1, TMED7 and Calnexin have all been described previously [10], [30], [33], [34], [41]. Goat polyclonal antibodies to PLIN3 (Tip47) and PLIN2 (ADRP) were purchased from Santa Cruz Biotechnologies Inc (Santa Cruz, Ca, USA). AlexaFluor 488 and AlexaFluor 568 secondary antibodies were purchased from Invitrogen (Carlsbad, CA). HRP-labeled polyclonal antibodies to rabbit IgG were purchased from Dianova (Hamburg, Germany). Protein A-HRP conjugate was purchased from Bio-Rad (Ontario, Canada).

### Indirect immunofluorescence and confocal microscopy

HeLa, McA-RH7777 and HepG2 cells were grown in DMEM supplemented with 10% FBS, penicillin (100 U/ml) and streptomycin (100 mg/ml). Lipid droplet formation was induced by adding 0.5 mM oleate (from 100 mM stock in ethanol) directly to media. In selected experiments, Bodipy 558/568 C_12_ was added at the same time as oleate to a final concentration of 3 µM in order to stain newly forming lipid droplets. cAMP was added cells to a final concentration of 37.5 µg/ml. Cells were processed for indirect immunofluorescence as described previously [34]. Post staining of neutral lipid droplets using 1 µg/ml Bodipy 493/503 was performed as described in [42]. All images were acquired using a LSM 700 series microscopy system (Carl Zeiss) fitted with a Plan-Apochromat 63x/1.40 Oil DIC objective in sequential scanning mode with the pinhole set to obtain an optical section of about 0.8 µm in both channels (approximately 1 Airy unit). For Alexa 488 and Bodipy 493/503, a 488 nm Argon ion laser was used and emitted fluorescence was filtered through a 505 to 530 nm band-pass. Bodipy 558/568 C_12_ and Alexa 568 was excited with a 561 nm DPSS laser and emitted fluorescence was filtered through a 585 to 690 nm band-pass. Tissue cryosections (see below) were cut from isopentane frozen tissue cubes at a thickness of approximately 6 microns and air dried for an hour then fixed in 4% paraformaldehyde solution for 30 minutes. Subsequent indirect immunofluorescence was performed as above. For live cell imaging, cells were grown and imaged as above in MatTek dishes (MatTek Corporation, Ashland, MA, USA) and imaged as above under normal tissue culture conditions (i.e. CO_2_, humidity and temperature). Prior to live cell imaging, cells were transfected with FuGENE HD according to manufacturers instructions (Roche Ltd, Laval, QC, Canada). EYFP was excited using a 512 nm laser line from an Argon ion laser and emission was filtered through an LP535 band-pass.

### siRNA transfection, lipid droplet staining and western blotting

RNA oligos were transfected with the siRNAMAX transfection reagent according to manufacturer instructions (Invitrogen, Carlsbad, CA). ARFGAP1 was knocked down using a sequence previously published [20], [43]. A scrambled sequence was used as a negative control. At 24 hours post transfection, cells were incubated with 0.5 mM oleate for 1 or 4 hours or left untreated. Cells were then fixed, imaged and processed as above. Lipid droplets were stained with Bodipy 493/503 (1 µg/ml) for 30 minutes. Western blotting was carried out as described previously [30].

### Image Analysis

Fluorescence levels were coded with a gray scale representing pixel intensity of 25-255. For semi-quantitation, in all cases background (pixel values outside the cells) was relatively low (<10% of the signal). Therefore, to minimize data manipulation, background subtraction was not performed. All images were processed and analyzed with ImageJ 1.45 s (National Institutes of Health, USA), where the mean intensities of the Golgi region and lipid images were measured to quantify the lipid droplets and the ARFGAP1 associated with the lipid droplets in each cell in the field of view. Quantitation of fluorescence was made from 150 to 350 cells for each experiment (10 sites/slide). Each experiment was performed at least three times. For quantification, images of HeLa, McA-RH7777 or HepG2 cells were processed with a binary segmentation algorithm. Briefly, after a sharpening, brightness/contrast adjustment (equal values for all images) and find edges, an Otsu's method thresholding step was run to automatically perform a reduction of a gray level image to a binary image. Watershed processing to identify solitary particles followed. Finally, lipid droplets (Bodipy 493/503 or 558/568) or Golgi region (TMED7 or ARFGAP1) were identified with the generic “analyze particles” function of the ImageJ software with the following settings: for the Golgi apparatus size from 100 to 10,000 pixels; and for the lipid droplets size from 1 to 2500 pixels and circularity from zero to one (final values were expressed in physical size square units); outlines as well as measurement results displayed and measurements on the edges excluded. The summary of the results was done through the ‘ROI’ (Region Of Interest) Manager that is a tool for working with multiple selections where areas to be evaluated can be specified editing out unwanted elements. For each image, the average particle size and cumulative measured area for all particles (“area”) were reported. The obtained information was used to calculate the ratio of lipid droplets per cells as a measure of lipid storage (“number of Bodipy-stained droplet/cell” or “of total area Bodipy-stained droplet/cell”) or ARFGAP1 associated with lipid droplets. All data were imported into Sigma Plot (SPSS Inc.). The Mann–Whitney rank sum test was used for statistical analysis. A *P* value of less than 0.05 was considered to be significant.

### Transmission electron microscopy

HeLa cells were fixed using a double fixation protocol with osmium and tannic acid [44]. Samples were dehydrated in graded ethanol series, and embedded in Epon 812 (Serva). After 48 h at 60°C, ultra-thin sections (60 nm) were cut and mounted on grids. Samples were examined on a LEO 912 OMEGA (Energy Filter Transmission Electron Microscope, Zeiss) at 120 kV accelerating voltage. Digital images were obtained through a side-mounted MegaView III TEM CCD camera.

### Immuno-gold transmission electron microscopy

Cryosections were prepared using an adaptation of the standard protocol developed by Tokuyasu [45]. Cells were fixed with a mix of 4% Formaldehyde and 0.1% Glutaraldehyde for 2 hours at 4°C and washed twice with PBS. A small volume of 1% gelatin in PBS was then added after which cells were removed from the dish using a rubber policeman cell scraper and transferred to a 1.5 ml Eppendorf tube and centrifuged for 30–60 sec at 1500 rpm. The 1% gelatin was then replaced with a 10% gelatin solution at 37°C. Cells were then resuspended and centrifuged as above after which excess gelatin was removed. Cells in gelatin were then solidified on ice for 30 minutes. At 4°C, the bottom of the Eppendorf tube was then cut off and 1-mm gelatin blocks were prepared from the pellet and incubated end over end in phosphate buffered 2.3 M sucrose overnight at 4°C. After trimming, ultrathin sections (75 nm) were prepared at −120°C. Sections were immediately transferred to a 1∶1 mixture of 2% methylcellulose and 2.3 M sucrose [46] and 2 µl of 4% uranyle acetate, and transferred to copper grids bearing a carbon-coated Formvar supporting film. Labeling was carried out at room temperature using specific antibodies and gold conjugates diluted in 0.5% fish skin gelatin in PBS. 10 nm colloidal gold-affiniPure Rabbit anti-Goat and 12 nm colloidal gold-affiniPure Goat anti-Rabbit IgG (H+L) were used to detect primary polyclonal antibodies. Sections were then embedded in a thin layer of 2% methylcellulose with 0.4% uranyl acetate, pH 4.0, air-dried, examined and photographed using a Philips/FEI Tecnai12 120 kV Transmission Electron Microscope equipped with an AMT XR80C CCD camera system.

### Processing of human liver tissue

Donor material was obtained through Transplant Quebec (http://transplantquebec.ca/en) and healthy resected liver material from patients undergoing liver surgery to remove liver metastasis. For resected material, removed tissue was immediately perfused with ice-cold Wisconsin solution, and within 30 minutes on ice, inspected by an on site pathologist, then processed for biobanking. For donor material, liver slices from discarded livers were perfused using ice-cold Wisconsin solution then processed for biobanking. For biobanking, liver tissue was diced into smaller pieces and placed for 5 minutes in isopentane precooled to −45°C in order to preserve morphology. Samples were then wrapped in tinfoil and kept frozen at −45°C until sectioned.

### Ethics statement

All handling and use of human patient and donor material was performed under IRB ethics approved protocols: "Liver Disease – Biobank" # 11-066-SDR and “Human Hepatic Tissue Organellar Isolation and Characterization" # 11-196-SDR filed at the McGill University Health Centre, Montreal, Canada in the name of Dr. Peter Metrakos. Patient and donor consent was obtained in written form for all human samples collected.

## Supporting Information

Figure S1 **Localization of ARFGAP1YFP in HeLa cells over the course of 40 minutes.** HeLa cells expressing ARFGAP1^YFP^ were imaged for 40 minutes to monitor the movement of ARFGAP1^YFP^-positive structures. The arrow shows the saltatory movement of a ring formed and lipid droplet-like ARFGAP1^YFP^-positive structure that moves in and out of the juxta nuclear Golgi region. Time of events is expressed in seconds. Images were recorded using a Zeiss LSM 510 META equipped with a 37°C climate chamber. Bar  = 10µm.
(TIF)

## References

[1] Guo Y, Walther TC, Rao M, Stuurman N, Goshima G, et al (2008) Functional genomic screen reveals genes involved in lipid-droplet formation and utilization. Nature 453: 657–661. 18408709 10.1038/nature06928 PMC2734507

[2] Beller M, Sztalryd C, Southall N, Bell M, Jackle H, et al (2008) COPI complex is a regulator of lipid homeostasis. PLoS Biol 6: e292. 19067489 10.1371/journal.pbio.0060292 PMC2586367

[3] Soni KG, Mardones GA, Sougrat R, Smirnova E, Jackson CL, et al (2009) Coatomer-dependent protein delivery to lipid droplets. J Cell Sci 122: 1834–1841. 19461073 10.1242/jcs.045849 PMC2684835

[4] Thiam AR, Antonny B, Wang J, Delacotte J, Wilfling F, et al (2013) COPI buds 60-nm lipid droplets from reconstituted water-phospholipid-triacylglyceride interfaces, suggesting a tension clamp function. Proc Natl Acad Sci U S A 110: 13244–13249. 23901109 10.1073/pnas.1307685110 PMC3746913

[5] Wilfling F, Thiam AR, Olarte MJ, Wang J, Beck R, et al (2014) Arf1/COPI machinery acts directly on lipid droplets and enables their connection to the ER for protein targeting. Elife 3: e01607. 24497546 10.7554/eLife.01607 PMC3913038

[6] Kahn RA (2009) Toward a model for Arf GTPases as regulators of traffic at the Golgi. FEBS Lett 583: 3872–3879. 19879269 10.1016/j.febslet.2009.10.066 PMC2787837

[7] Kahn RA, Randazzo P, Serafini T, Weiss O, Rulka C, et al (1992) The amino terminus of ADP-ribosylation factor (ARF) is a critical determinant of ARF activities and is a potent and specific inhibitor of protein transport. J Biol Chem 267: 13039–13046. 1618801

[8] Balch WE, Kahn RA, Schwaninger R (1992) ADP-ribosylation factor is required for vesicular trafficking between the endoplasmic reticulum and the cis-Golgi compartment. J Biol Chem 267: 13053–13061. 1618803

[9] Donaldson JG, Kahn RA, Lippincott-Schwartz J, Klausner RD (1991) Binding of ARF and beta-COP to Golgi membranes: possible regulation by a trimeric G protein. Science 254: 1197–1199. 1957170 10.1126/science.1957170

[10] Lanoix J, Ouwendijk J, Lin CC, Stark A, Love HD, et al (1999) GTP hydrolysis by arf-1 mediates sorting and concentration of Golgi resident enzymes into functional COP I vesicles. EMBO J 18: 4935–4948. 10487746 10.1093/emboj/18.18.4935 PMC1171565

[11] Malsam J, Gommel D, Wieland FT, Nickel W (1999) A role for ADP ribosylation factor in the control of cargo uptake during COPI-coated vesicle biogenesis. FEBS Lett 462: 267–272. 10622709 10.1016/s0014-5793(99)01543-4

[12] Pepperkok R, Whitney JA, Gomez M, Kreis TE (2000) COPI vesicles accumulating in the presence of a GTP restricted arf1 mutant are depleted of anterograde and retrograde cargo. J Cell Sci 113 (Pt 1): 135–144. 10.1242/jcs.113.1.135 10591632

[13] Cockcroft S, Thomas GM, Fensome A, Geny B, Cunningham E, et al (1994) Phospholipase D: a downstream effector of ARF in granulocytes. Science 263: 523–526. 8290961 10.1126/science.8290961

[14] Brown HA, Gutowski S, Moomaw CR, Slaughter C, Sternweis PC (1993) ADP-ribosylation factor, a small GTP-dependent regulatory protein, stimulates phospholipase D activity. Cell 75: 1137–1144. 8261513 10.1016/0092-8674(93)90323-i

[15] Godi A, Pertile P, Meyers R, Marra P, Di Tullio G, et al (1999) ARF mediates recruitment of PtdIns-4-OH kinase-beta and stimulates synthesis of PtdIns(4,5)P2 on the Golgi complex. Nat Cell Biol 1: 280–287. 10559940 10.1038/12993

[16] Boman AL, Zhang C, Zhu X, Kahn RA (2000) A family of ADP-ribosylation factor effectors that can alter membrane transport through the trans-Golgi. Mol Biol Cell 11: 1241–1255. 10749927 10.1091/mbc.11.4.1241 PMC14844

[17] Dell'Angelica EC, Puertollano R, Mullins C, Aguilar RC, Vargas JD, et al (2000) GGAs: a family of ADP ribosylation factor-binding proteins related to adaptors and associated with the Golgi complex. J Cell Biol 149: 81–94. 10747089 10.1083/jcb.149.1.81 PMC2175099

[18] Nakamura N, Akashi T, Taneda T, Kogo H, Kikuchi A, et al (2004) ADRP is dissociated from lipid droplets by ARF1-dependent mechanism. Biochem Biophys Res Commun 322: 957–965. 15336557 10.1016/j.bbrc.2004.08.010

[19] Nakamura N, Banno Y, Tamiya-Koizumi K (2005) Arf1-dependent PLD1 is localized to oleic acid-induced lipid droplets in NIH3T3 cells. Biochem Biophys Res Commun 335: 117–123. 16054594 10.1016/j.bbrc.2005.07.050

[20] Asp L, Kartberg F, Fernandez-Rodriguez J, Smedh M, Elsner M, et al (2009) Early stages of Golgi vesicle and tubule formation require diacylglycerol. Mol Biol Cell 20: 780–790. 19037109 10.1091/mbc.E08-03-0256 PMC2633395

[21] Fernandez-Ulibarri I, Vilella M, Lazaro-Dieguez F, Sarri E, Martinez SE, et al (2007) Diacylglycerol is required for the formation of COPI vesicles in the Golgi-to-ER transport pathway. Mol Biol Cell 18: 3250–3263. 17567948 10.1091/mbc.E07-04-0334 PMC1951743

[22] Antonny B, Huber I, Paris S, Chabre M, Cassel D (1997) Activation of ADP-ribosylation factor 1 GTPase-activating protein by phosphatidylcholine-derived diacylglycerols. J Biol Chem 272: 30848–30851. 9388229 10.1074/jbc.272.49.30848

[23] Bigay J, Gounon P, Robineau S, Antonny B (2003) Lipid packing sensed by ArfGAP1 couples COPI coat disassembly to membrane bilayer curvature. Nature 426: 563–566. 14654841 10.1038/nature02108

[24] Cukierman E, Huber I, Rotman M, Cassel D (1995) The ARF1 GTPase-activating protein: zinc finger motif and Golgi complex localization. Science 270: 1999–2002. 8533093 10.1126/science.270.5244.1999

[25] Lee SY, Yang JS, Hong W, Premont RT, Hsu VW (2005) ARFGAP1 plays a central role in coupling COPI cargo sorting with vesicle formation. J Cell Biol 168: 281–290. 15657398 10.1083/jcb.200404008 PMC2171589

[26] Bai M, Gad H, Turacchio G, Cocucci E, Yang JS, et al (2011) ARFGAP1 promotes AP-2-dependent endocytosis. Nat Cell Biol 13: 559–567. 21499258 10.1038/ncb2221 PMC3087831

[27] Stafa K, Trancikova A, Webber PJ, Glauser L, West AB, et al (2012) GTPase activity and neuronal toxicity of Parkinson's disease-associated LRRK2 is regulated by ArfGAP1. PLoS Genet 8: e1002526. 22363216 10.1371/journal.pgen.1002526 PMC3280333

[28] Xiong Y, Yuan C, Chen R, Dawson TM, Dawson VL (2012) ArfGAP1 is a GTPase activating protein for LRRK2: reciprocal regulation of ArfGAP1 by LRRK2. J Neurosci 32: 3877–3886. 22423108 10.1523/JNEUROSCI.4566-11.2012 PMC3319331

[29] Aoe T, Cukierman E, Lee A, Cassel D, Peters PJ, et al (1997) The KDEL receptor, ERD2, regulates intracellular traffic by recruiting a GTPase-activating protein for ARF1. EMBO J 16: 7305–7316. 9405360 10.1093/emboj/16.24.7305 PMC1170331

[30] Lanoix J, Ouwendijk J, Stark A, Szafer E, Cassel D, et al (2001) Sorting of Golgi resident proteins into different subpopulations of COPI vesicles: a role for ArfGAP1. J Cell Biol 155: 1199–1212. 11748249 10.1083/jcb.200108017 PMC2199348

[31] Ou WJ, Cameron PH, Thomas DY, Bergeron JJ (1993) Association of folding intermediates of glycoproteins with calnexin during protein maturation. Nature 364: 771–776. 8102790 10.1038/364771a0

[32] Diaz E, Pfeffer SR (1998) TIP47: a cargo selection device for mannose 6-phosphate receptor trafficking. Cell 93: 433–443. 9590177 10.1016/s0092-8674(00)81171-x

[33] Duden R, Griffiths G, Frank R, Argos P, Kreis TE (1991) Beta-COP, a 110 kd protein associated with non-clathrin-coated vesicles and the Golgi complex, shows homology to beta-adaptin. Cell 64: 649–665. 1840503 10.1016/0092-8674(91)90248-w

[34] Fullekrug J, Suganuma T, Tang BL, Hong W, Storrie B, et al (1999) Localization and recycling of gp27 (hp24gamma3): complex formation with other p24 family members. Mol Biol Cell 10: 1939–1955. 10359607 10.1091/mbc.10.6.1939 PMC25391

[35] Jungas RL (1966) Role of cyclic-3',5'-amp in the response of adipose tissue to insulin. Proc Natl Acad Sci U S A 56: 757–763. 16591373 10.1073/pnas.56.2.757 PMC224437

[36] Bartz R, Zehmer JK, Zhu M, Chen Y, Serrero G, et al (2007) Dynamic activity of lipid droplets: protein phosphorylation and GTP-mediated protein translocation. J Proteome Res 6: 3256–3265. 17608402 10.1021/pr070158j

[37] Takashima K, Saitoh A, Hirose S, Nakai W, Kondo Y, et al (2011) GBF1-Arf-COPI-ArfGAP-mediated Golgi-to-ER transport involved in regulation of lipid homeostasis. Cell Struct Funct 36: 223–235. 22185782 10.1247/csf.11035

[38] Gubern A, Barcelo-Torns M, Casas J, Barneda D, Masgrau R, et al (2009) Lipid droplet biogenesis induced by stress involves triacylglycerol synthesis that depends on group VIA phospholipase A2. J Biol Chem 284: 5697–5708. 19117952 10.1074/jbc.M806173200

[39] Bostrom P, Andersson L, Rutberg M, Perman J, Lidberg U, et al (2007) SNARE proteins mediate fusion between cytosolic lipid droplets and are implicated in insulin sensitivity. Nat Cell Biol 9: 1286–1293. 17922004 10.1038/ncb1648

[40] Chen JL, Xu W, Stamnes M (2005) In vitro reconstitution of ARF-regulated cytoskeletal dynamics on Golgi membranes. Methods Enzymol 404: 345–358. 16413281 10.1016/S0076-6879(05)04030-9

[41] Wada I, Rindress D, Cameron PH, Ou WJ, Doherty JJ 2nd, et al (1991) SSR alpha and associated calnexin are major calcium binding proteins of the endoplasmic reticulum membrane. J Biol Chem 266: 19599–19610. 1918067

[42] Gocze PM, Vahrson HW, Freeman DA (1994) Serum levels of squamous cell carcinoma antigen and ovarian carcinoma antigen (CA 125) in patients with benign and malignant diseases of the uterine cervix. Oncology 51: 430–434. 8052484 10.1159/000227378

[43] Frigerio G, Grimsey N, Dale M, Majoul I, Duden R (2007) Two human ARFGAPs associated with COP-I-coated vesicles. Traffic 8: 1644–1655. 17760859 10.1111/j.1600-0854.2007.00631.x PMC2171037

[44] Simionescu N, Simionescu M (1976) Galloylglucoses of low molecular weight as mordant in electron microscopy. I. Procedure, and evidence for mordanting effect. J Cell Biol 70: 608–621. 783172 10.1083/jcb.70.3.608 PMC2109842

[45] Tokuyasu KT (1973) A technique for ultracryotomy of cell suspensions and tissues. J Cell Biol 57: 551–565. 4121290 10.1083/jcb.57.2.551 PMC2108989

[46] Liou W, Geuze HJ, Slot JW (1996) Improving structural integrity of cryosections for immunogold labeling. Histochem Cell Biol 106: 41–58. 8858366 10.1007/BF02473201

[47] Liu W, Duden R, Phair RD, Lippincott-Schwartz J (2005) ArfGAP1 dynamics and its role in COPI coat assembly on Golgi membranes of living cells. J Cell Biol 168: 1053–1063. 15795316 10.1083/jcb.200410142 PMC2171832

