# Supplementary figures and images for: ARFGAP1 Is Dynamically Associated with Lipid Droplets in Hepatocytes
Source: PLoS One. 2014 Nov 14;9(11):e111309. doi: 10.1371/journal.pone.0111309 (PMC4232254; doi:10.1371/journal.pone.0111309)

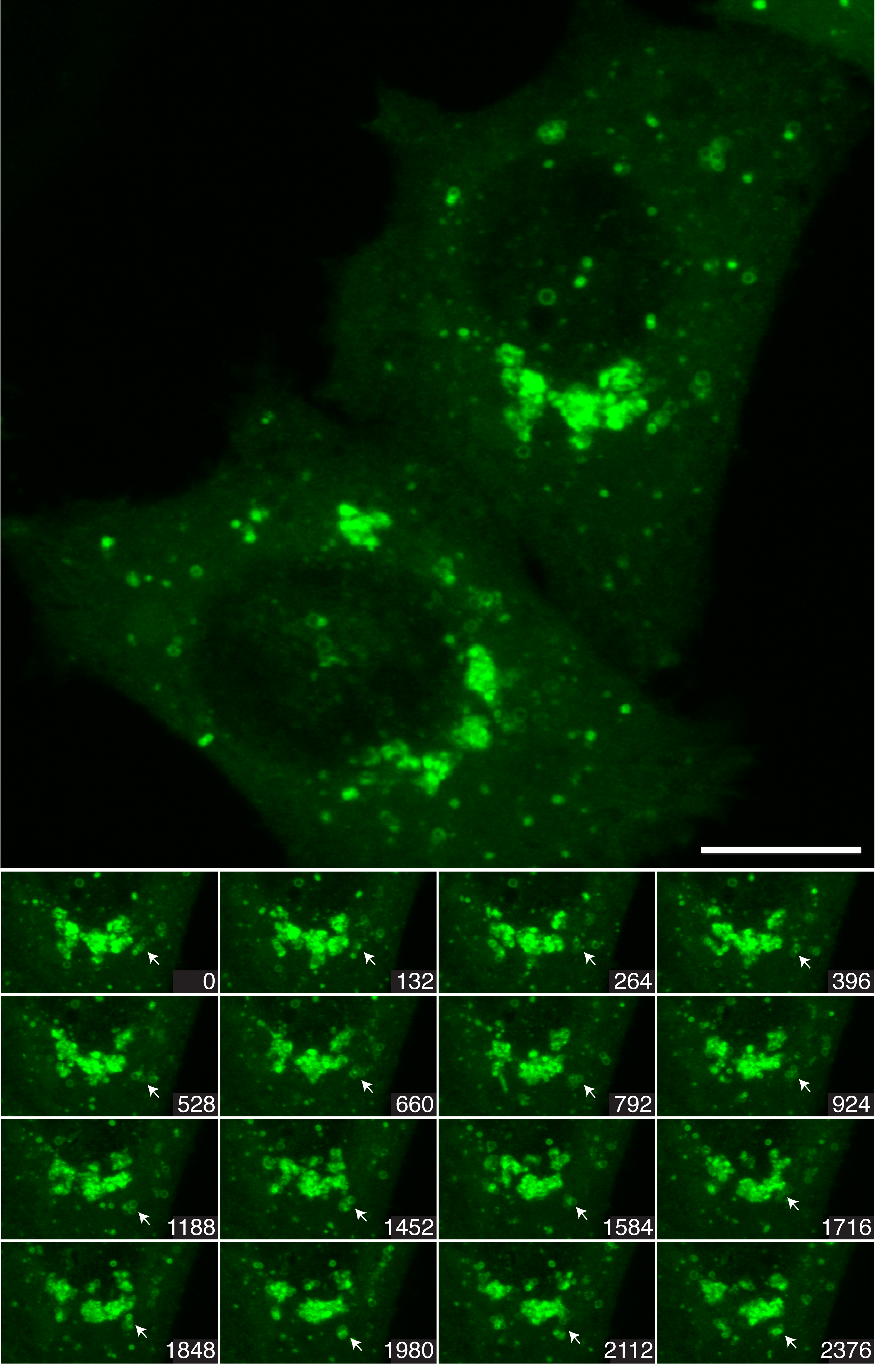

Supplement: Figure S1 — Localization of ARFGAP1YFP in HeLa cells over the course of 40 minutes. HeLa cells expressing ARFGAP1YFP were imaged for 40 minutes to monitor the movement of ARFGAP1YFP-positive structures. The arrow shows the saltatory movement of a ring formed and lipid droplet-like ARFGAP1YFP-positive structure that moves in and out of the juxta nuclear Golgi region. Time of events is expressed in seconds. Images were recorded using a Zeiss LSM 510 META equipped with a 37°C climate chamber. Bar = 10µm. (TIF) [file pone.0111309.s001.tif]
